# Analysis of Effectiveness of Individual and Group Trauma-Focused Interventions for Female Victims of Intimate Partner Violence

**DOI:** 10.3390/ijerph18041952

**Published:** 2021-02-17

**Authors:** María Crespo, María Arinero, Carmen Soberón

**Affiliations:** Department Personality, Assessment and Clinical Psychology, Universidad Complutense de Madrid, 28223 Madrid, Spain; arinerom@yahoo.es (M.A.); c.soberon@ucm.es (C.S.)

**Keywords:** intimate partner violence, psychological treatment, randomized controlled trial, posttraumatic stress, effectiveness

## Abstract

Group psychological programs for intimate partner violence (IPV) survivors would seem particularly useful since they contribute to interrupting women’s isolation and have cost-effectiveness advantage. This study aims to analyze whether the effectiveness of group interventions for female survivors of IPV is equivalent to that of the individual format. A cognitive-behavioral trauma-focused intervention program was applied in eight weekly sessions in Madrid (Spain) to IPV female survivors with significant posttraumatic symptoms that were randomly assigned to the individual (*n* = 25) or group (*n* = 28) intervention format. Measures of posttraumatic stress (Severity of Posttraumatic Stress Disorder Symptoms Scale), depression (Beck Depression Inventory), anxiety (Beck Anxiety Inventory), self-esteem (Rosenberg’s Scale) and social support were analyzed at pre-treatment, post-treatment, and 1-, 3-, 6- and 12-months follow-ups. A total of 28.3% of women dropped out, without significant format differences. Intervention (both formats) had significant improvements with large effect sizes in posttraumatic stress (η^2^_p_ = 0.56), depression (η^2^_p_ = 0.45), anxiety (η^2^_p_ = 0.41) and self-esteem (η^2^_p_ = 0.26) that maintained in follow-ups (*p* < 0.001), without significant differences between formats. Both intervention formats had different evolutions for depression and anxiety (*p* < 0.05), with better effects in the individual format at the first post-test measurements, but the differences tended to disappear over time. Intervention was effective in improving social support, with no significant differences between formats. All in all, both formats showed similar effectiveness. The group format could be an alternative when applying psychological interventions for female IPV survivors, since it would maintain good cost-effectiveness balance, mainly in the long-term.

## 1. Introduction

Intimate partner violence (IPV) is currently one of the major public health problems around the world; according to the systematic review on violence against women published by the World Health Organization [1], the global prevalence of physical and/or sexual intimate partner violence among all ever-partnered women was 30.0%. Similarly, data from the nationally representative Spanish Survey on Violence Against Women 2015 suggest that 30.7% of Spanish women aged 16 and above have experienced at least one type of IPV during their lives, and 13.2% reported IPV in the 12 months prior to the assessment [2], while according to the 2015 National Intimate Partner and Sexual Violence Survey, 33.6% of women in the United States have experienced rape, physical violence, and/or stalking by an intimate partner in their lifetime, and 5.2% in the last 12-months [3].

Women victims of IPV are exposed to chronic and often extreme maltreatment that can lead to a broad range of physical, social and psychological outcomes. In their systematic review, Langdon et al. [4] concluded that IPV can have more adverse effects on mental health in comparison to non-IPV victims and victims of other traumatic events, being Posttraumatic Stress Disorder (PTSD), depression and anxiety, which are the most consistent mental health outcomes associated with IPV across the studies. Actually, the recent WHO World Mental Health Survey [5] posed the physical abuse by a partner as one of the traumatic events that conveys the greatest risk for PTSD. Likewise, IPV has also been associated with other relevant variables, such as suicidal ideation [6,7], alcohol and substances use [8], poor health status and self-perceived health [2,6], somatization [4], and functional impairments [9].

On the basis of increasing rates of IPV and its potential consequences, in the last twenty years, a considerable number of interventions have been designed or modified specifically for IPV survivors. In a systematic review and meta-analysis about short-term interventions for survivors of IPV, Arroyo et al. [10] found that most interventions analyzed were effective compared to not receiving treatment, achieving large effect sizes in PTSD, self-esteem, depression, general distress and life functioning. Regarding treatment type, largest effects sizes were found for cognitive behavioral therapies (CBT) and interpersonal therapies specifically tailored to IPV survivors. In addition, individually delivered interventions produced significantly stronger outcomes than group delivered interventions. However, authors noted that some methodological weaknesses in analyzed studies could have biased their findings.

Individual interventions may lead to a closer attention to survivors, and therefore could be more tailored and targeted to the specific needs of women [10]. In addition, some IPV survivors could prefer individual interventions because they may feel worse sharing their problems in a therapeutic group [11]. Furthermore, the implementation of some intervention techniques, such as exposure therapy, could be more problematic in the group delivery format [12]. However, regarding cost-effectiveness, it may be difficult to deliver individual interventions in settings where economic and human sources are limited [13]. Likewise, group interventions may convey additional benefits to women victims of IPV from a social perspective; the group could be a source of social learning where IPV survivors could change and acquire skills through interactions with other women facing similar situations [13,14]; additionally, the group may also provide social support [13,14], a relevant protective factor against IPV mental health outcomes [6]. In this sense group programs would be adequate to interrupt women’s isolation and would seem particularly useful for long term or chronic posttraumatic symptoms [15]. Furthermore, the group format is often chosen for delivering interventions due to its cost-effectiveness advantage [15].

Despite the clinical relevance of the intervention delivery type, the only study that has directly compared the effectiveness of an intervention program tailored to female IPV survivors in the individual versus group format was by Fernández-Velasco’s [12]. In this study, which included 95 Spanish women victims of IPV, both intervention delivery types have proven to be effective to treat PTSD, depression, self-esteem and general maladjustment, although the individual format was superior to group intervention, mainly at 6- and 12-months follow-ups. However, as the author noted, the fact that two different therapists implemented each intervention format could have skewed these findings. In addition, this study only includes IPV survivors who met the criteria for PTSD diagnosis, which may limit the generalizability of these findings to IPV victims who may manifest sub-clinical levels of posttraumatic symptoms that are nonetheless seriously disabling and may require psychological intervention. Finally, regarding social variables, although this study included a measure of maladjustment associated with overall symptomatology, it did not specifically associate this impairment to posttraumatic symptoms, and it did not assess other important social variables in IPV survivors such as social support.

The purpose of the present study was to explore the differential effectiveness of individual and group intervention for female survivors of IPV. Therefore, it involves a comparison of individual vs. group formats of a brief CBT trauma-focused intervention program that has previously showed its effectiveness for women victims of IPV with sub-clinical PTSD symptoms (cf. [16]).

## 2. Materials and Methods

### 2.1. Objectives and Hypotheses

To establish the differential effectiveness of the individual and group delivery formats, the study analyzes format differences in adherence to treatment, as well as efficacy and clinical significance of the improvements achieved in the posttreatment and at 1-, 3-, 6- and 12 months follow-ups. Primary outcome variables were overall posttraumatic symptoms, and associated symptoms of depression and anxiety. Other variables relevant to battered women emotional status (namely self-esteem) were considered to be secondary variables. Moreover, in order to assess the specific effects of both formats on social issues, we also examined variables such as social and family support.

Based on previous research, it was hypothesized that (1) adherence to treatment would be higher in the individual format than in the group one; (2) both intervention formats would reduce posttraumatic symptoms, depression and anxiety, and increase self-esteem, and would produce clinically significant changes; (3) individual format effect on outcome variables would be superior; and (4) group format would show a higher effect on social issues.

### 2.2. Participants

Participants were recruited from several organizations and institutions in the area of Madrid (Spain), which offered programs for women that suffer IPV. All the women that initially attended these services for attention because of IPV were assessed to establish the eligibility criteria: being woman 18 years of age or older, having suffered violence by a male intimate partner, presenting posttraumatic symptoms in the Severity of Posttraumatic Stress Disorder Symptoms Scale [17] without meeting all the diagnostic criteria for PTSD according to the Diagnostic and Statistical Manual of Mental Disorders, 4th ed. revised [18]; and receiving no other current treatment. When a woman presented symptoms that met all the diagnostic criteria for PTSD, she was derived to a parallel treatment specifically designed for battered women with PTSD that was carried out by a different research team. Exclusion criteria included conditions that could prevent compliance with the treatment (namely, abuse of alcohol or drugs, cognitive impairment or illiteracy in Spanish).

**Figure 1:**
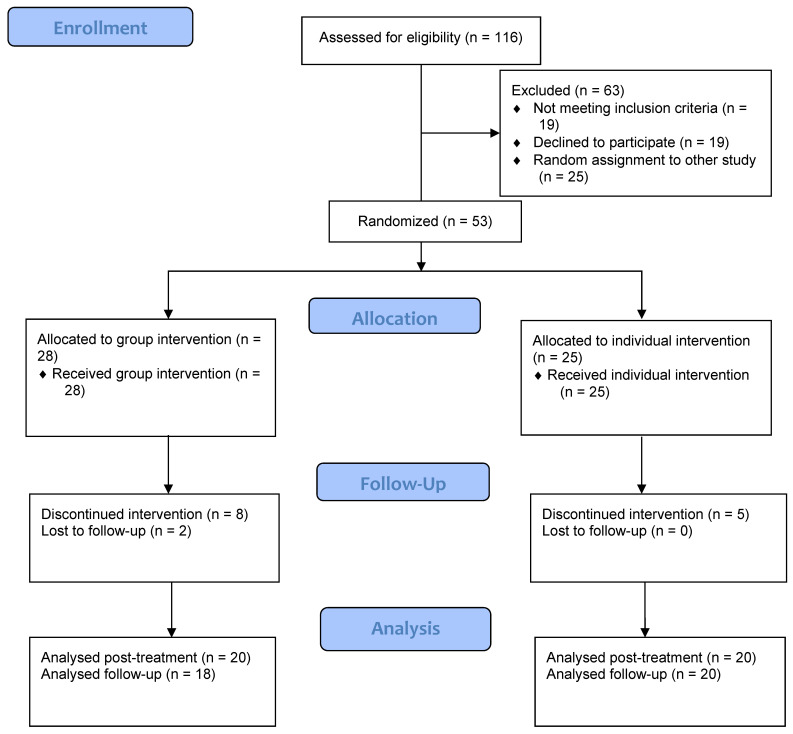
CONSORT participants flow diagram.

One-hundred and sixteen women were initially assessed. Nineteen were excluded for meeting PTSD diagnostic criteria and 19 rejected the treatment for different reasons (schedule problems, change of residence, etc.). Consequently, 78 accepted the treatment; nevertheless, this study reports the data of 53, since the other 25 were included in a former phase of the project [16]. Their participation in the treatment was voluntary and was always carried out after the women were informed of the goal of the study and guaranteeing the confidentiality of the information provided. Figure 1 illustrates the participants CONSORT (Consolidated Standards of Reporting Trials) diagram.

### 2.3. Design

A multigroup (two groups) experimental design was employed with repeated pre- and post-measures (2 months later) and follow-up measures taken at 1, 3, 6 and 12 months after the end of the treatment. The independent variable was the psychological intervention (a brief multicomponent CBT program), with two levels according to their delivering format: individual vs. group.

Participants were assigned to each experimental condition by a balanced random process using the randomization.com computer program (cf. http://www.randomization.com (accessed on 10 December 2020)). In this way, 28 participants were assigned to the group format and 25 to the individual one. The study protocol was approved by the Faculty Ethics Committee (number 2016/17-022) and it was conducted following CONSORT recommendations [19].

### 2.4. Variables and Measures

#### 2.4.1. Demographic Variables and History and Features of Violence

A standardized interview [20] assessed background information (e.g., age, subjective social class, educational level, civil status, etc.), and information about the history and features of violence, considering the type, duration, frequency, need of help and attention (legal, medical and psychiatric/psychological), and perception of support received (familiar and social). The interview included the Cut-down, Annoyed, Guilty and Eye-opener- CAGE questionnaire ([21]; Spanish translation by Fonseca del Pozo et al. [22]) to assess the possibility of abusive consumption of alcohol. In Spanish samples, the CAGE has shown a sensitivity of 96% and a specificity of 100% at a cutoff point of 1 [23].

#### 2.4.2. Outcome Variables

Severity of Posttraumatic Stress Disorder Symptoms Scale (in Spanish Escala de Gravedad de Síntomas del Trastorno de Estrés Postraumático, EGS; Echeburúa et al. [17]) was used to assess the severity of posttraumatic symptoms ranging from 0 to 51 (higher scores showing more severe symptoms). The test-retest reliability coefficient of this scale was 0.89 (*p* ≤ 0.001) with a 4-week interval, and its internal consistency index (Cronbach’s alpha) was 0.92. The global score reaches a sensitivity of 100% and a specificity of 93.7% at a cut-off point of 15 to 16.

Beck Depression Inventory (BDI-II; Beck et al. [24], in the Spanish version by Sanz et al. [25]) identifies the global level of depression and the changes over time. The scores range was 0–63 (higher scores showing more severe symptoms). The published Spanish adaptation of the BDI-II [25] proposes a cut-off point of 13 to differentiate the minimum or slight levels of depression from moderate or severe levels. The Spanish version of the inventory has shown good internal consistency: 0.90 with subclinical samples [26] and 0.89 with patients presenting diverse disorders [27].

Beck Anxiety Inventory (BAI; Beck et al. [28], in the Spanish version by Sanz and Navarro [29]) assesses anxiety symptoms, focusing particularly on the physiological symptoms (66.7%). The remaining items refer to affective (14.3%) and cognitive aspects (19%). The scores range was 0–63 (higher scores showing more severe symptoms). According to Sanz [30], a cut-off point of 13 differentiates normal or slight levels of anxiety from moderate to severe levels. The internal consistency of the Spanish version was satisfactory (α = 0.88).

Rosenberg’s Self-Esteem Scale (Rosenberg [31]; Spanish version by Echeburúa and Corral [32]) assesses women’s levels of self-esteem, which is a person’s feelings of self-satisfaction and self-acceptance. The scores range was 10–40 (higher scores showing higher self-esteem) and a cut-off point of 29 differentiates low from high self-esteem. Its internal consistency was 0.81 and its discriminant validity was adequate.

All these instruments, except the standardized interview that was applied only in pre-treatment assessment, were readministered at post-treatment and the follow-ups. Additionally, the Client Satisfaction Questionnaire (CSQ-8; Larsen et al. [33]; Spanish version by Echeburúa and Corral [32]) was introduced in post-treatment assessment to evaluate the quality of the program and the women’s satisfaction with the treatment. The scores range was 8–32, showing higher scores greater satisfaction.

### 2.5. Procedure

The treatment used in this study was a multicomponent cognitive-behavioral program based on Rincón and Labrador’s work [20] that, on the basis of the specific needs of this population and through a review of prior treatment studies, includes the following modules: (a) psycho-education, providing the participants information about IPV and its consequences for the victim; (b) exercises to control arousal by diaphragmatic breathing (focused on hyper-alertness symptoms); (c) planning to increase pleasant activities as a way to improve mood; (d) specific techniques to improve self-esteem; (e) restructuring of biased cognitions; (f) increase of skills for an independent life (by training in problem-solving); (g) exposure techniques in imagination focused on the memory of IPV situations to address re-experiencing and avoidance responses; and (h) relapse prevention.

All in all, the program consisted of 8 weekly sessions (which implies a total program duration of two months). The first session included presentations and the establishment of the treatment rules besides some information about the violence and about the intervention format. The other sessions started with a brief “check in” and review of the homework, followed by some new elements (cognitive-behavioral skills) to discuss and practice, and ended with 5 min of diaphragmatic breathing. The specific content for each session was as follows: (1) psycho-education (session 1); (2) exercises of diaphragmatic breathing (sessions 1–7); (3) planning to increase pleasant activities (sessions 2–3); (4) techniques to improve self-esteem (sessions 2–3); (5) cognitive restructuring of biased cognitions (sessions 2–4); (6) training in problem-solving (sessions 4–5); and (7) exposure techniques (sessions 6–7). Finally, session 8 was focused on relapse prevention.

Sessions lasted 60 min for the individual format and 90 min for the group version. Group format intervention was delivered in groups of 3–5 women. Between sessions, the women of both experimental groups received written material that outlined the topics from the session, as well as exercises to be done as homework. Women’s performance of trained skills was monitored by counting the number of completed homework sheets returned by each woman over the duration of the study. Assessment was performed individually before treatment and then after treatment and the four follow-ups in sessions of about 90 min carried out by the same person.

In order to avoid potential bias, both intervention formats were conducted by the same therapist. Likewise, the intervention protocol and homework sheets were manualized to ensure the homogeneity and replicability. Further details about the program can be found elsewhere [16,34]. The manual of the intervention protocol as well as all the therapeutic materials are available upon request to the authors.

### 2.6. Data Analysis

Analyses were performed using an intent-to-treat (ITT) approach. Little’s MCAR test [35] showed that the data were missing completely at random (MCAR), so that the maximum likelihood estimation was applied for replacement. Kolmogorov-Smirnov tests and Levene’s test for equality of variances were applied to check the distribution of the data for the pretreatment measure of the outcome variables in the two experimental conditions. Since the results confirmed normal distribution and equality of variances, independent samples *t*-test, Chi-square test and Fisher exact test were used to verify the homogeneity of the groups, using Cohen’s *d* and phi coefficient (Φ) as measures of the effect sizes. 2 (Group) x 6 (Time) repeated measures analysis of variance (ANOVA_s_) were used to assess changes in clinical variables and psychosocial functioning, computing pairwise differences using Bonferroni correction. However, in those outcome variables in which the groups were not initially homogeneous, one-factor ANCOVA_s_ were performed to analyze between-subjects differences controlling pretreatment levels. Partial eta-squared (η^2^_p_) was calculated to assess effect sizes for within and between-subjects’ comparisons. All effect sizes were interpreted using the benchmarks provided by Cohen [36] (i.e., Cohen’s d: small < 0.50, medium > 0.50 and < 0.80, and large > 0.80; Φ: small < 0.30, medium > 0.30 and < 0.50, and large > 0.50; η^2^_p_: small < 0.06, medium > 0.06 and < 0.14, and large > 0.14).

In addition, McNemar’s tests were performed to determine the pre-post changes in the percentage of women with clinically significant scores and social variables in the two intervention formats.

## 3. Results

### 3.1. Adherence to Treatment

A total of 15 (28.30%) women dropped out of treatment, 13 during the sessions and 2 at follow-up. While the percentage of dropouts was higher in the group format than in the individual one (35.71% vs. 20%, respectively), this difference was not statistically significant (χ^2^(1) = 0.92, *p* = 0.336, Φ = 0.174). Moreover, all the dropouts in the individual intervention occurred during the first four sessions, whereas two women in group intervention (7.14%) dropped-out at follow-up.

Regarding attendance at the sessions, significant differences were found (χ^2^(1) = 6.12, *p* < 0.05). As might be expected, all the women in the individual format attended all the sessions (since this was tailored to their availability), compared to 80% of attendance in the group intervention. There were no significant differences between groups in the performing homework, although it was higher for the individual intervention (95.90% vs. 89.24%).

Women’s satisfaction with the program was high in both formats (individual M = 31; SD = 0.97; group M = 30.2; SD = 1.47), without significant differences between them in the total CSQ-8 score or in any of the eight assessed issues.

### 3.2. Sample Characteristics and Group Homogeneity

The mean age of the participants was 39.17 (*SD* = 10.19) years, ranging from 23 to 65. Most of them belonged to the middle social class (50.9%) and had completed primary (35.8%) or secondary studies (39.6%). 49.1% worked outside home, whereas 32.1% reported being housewives. More than one half of the samples (50.9%) were separated or underwent separation procedures, although about one third (30.2%) still lived with their aggressor at the time of the assessment, and 37.7% depended on him economically.

Regarding their history of maltreatment, it can be considered prolonged, with a mean of more than 11 years (M = 11.26; SD = 10.57), and of daily frequency in the past month for about 45% of the sample, with 45.3% classifying the current status of their problem as “the worst moment.” Regarding the kind of maltreatment suffered, the most frequent (58.5%) was the combination of physical and psychological abuse; a great majority (96.23%) had suffered psychological abuse; physical abuse was also very common (66.04%); and sexual abuse was reported much less frequently (7.55%), and always in combination with psychological and/or physical abuse.

Sixty-four percent had presented charges about their situation, and about the half of the participants (52.8%) had to leave their home because of the violence. More than one third of the samples had received medical, psychological, or psychiatric attention because of the violence, and over 50% were taking medication (mainly antidepressant and anxiolytic drugs). In contrast, most of the women felt supported, principally by their families (79.2%), but also at a social (60.4%) and legal level (52.8%).

With regard to their emotional status, although none of the women met the diagnostic criteria for PTSD, as imposed by the inclusion criteria, 50.9% presented posttraumatic symptomatology above the EGS cut-off point, which indicates the clinical severity of these symptoms.

The women’s mean depression score can be considered severe (according to the cut-off points established for the Spanish version of the scale), whereas the mean anxiety score was considered moderate–severe. Moreover, at the time of assessment, about half of the participants (50.9%) admitted having had suicide ideation. Likewise, the level of these women’s self-esteem can be considered medium, and 50.9% of the interviewees were below this cut-off point. In contrast to these data, the mean alcohol consumption score was minimum, practically null for the total sample.

In order to assess groups homogeneity, group differences in sociodemographic variables, violence history, psychological health, and social issues were analyzed. As can be seen in Table 1, there were no statistically significant differences between the groups in sociodemographic variables and violence history. Regarding outcome variables, significant differences were observed in the mean levels of depression and anxiety, which were higher in the individual intervention. While the severity of the posttraumatic symptoms was also higher in the individual condition, differences did not reach statistical significance. In addition, participants in individual intervention also showed significantly lower scores in self-esteem. Finally, participants in the group format reported higher family and social support, although differences did not reach statistical significance.

### 3.3. Treatment Effectiveness

**Figure 2:**
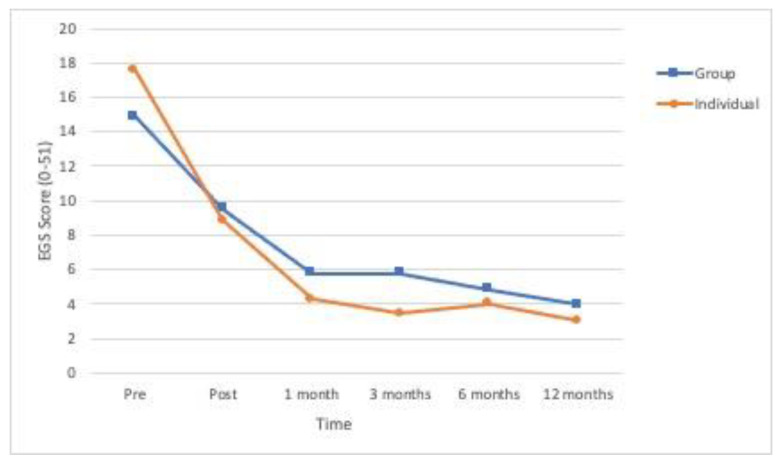
Evolution over time of the levels of posttraumatic symptoms (Severity of Posttraumatic Stress Disorder Symptoms Scale -EGS) in the two experimental conditions (*n* = 53).

**Figure 3:**
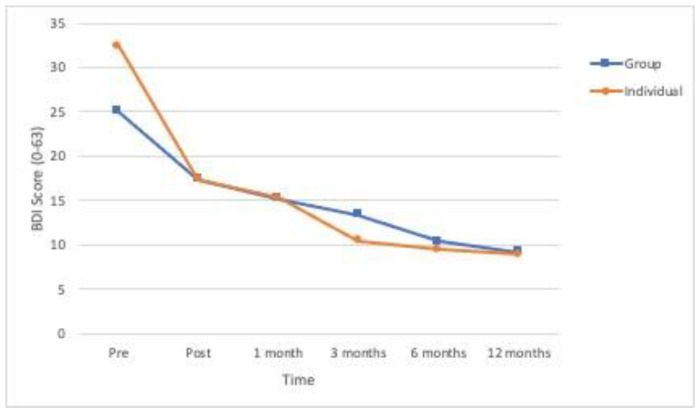
Evolution over time of the levels of depression (Beck Depression Inventory-BDI) in the two experimental conditions (*n* = 53).

**Figure 4:**
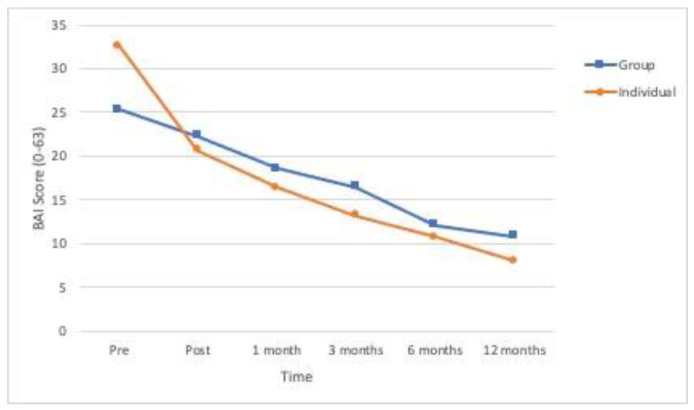
Evolution over time of the levels of anxiety (Beck Anxiety Inventory-BAI) in the two experimental conditions (*n* = 53).

**Figure 5:**
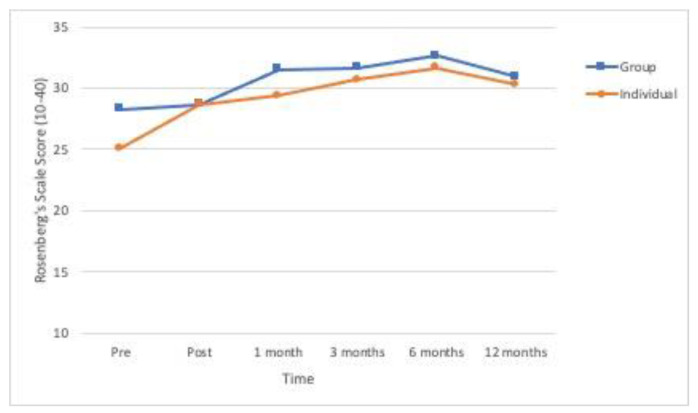
Evolution over time of the levels of self-esteem (Rosenberg’s Self-Esteem Scale) in the two experimental conditions (*n* = 53).

All the primary outcome variables and the secondary variable showed significant differences in time (Table 2). As can be seen in Figure 2, Figure 3 and Figure 4, the means for posttraumatic symptoms, depression and anxiety show a pronounced and significant decrease between the baseline or pre-treatment values and the respective posttreatment values and these improvements are more or less sustained at the follow-ups (*p* < 0.01). In the case of self-esteem, the significant improvement (*p* < 0.01) delays until 1-month follow-up and is sustained from that moment (see Figure 5). All the effect sizes for time were large.

More interestingly for the objectives of this study (Table 2), repeated measure ANOVAs did not reveal significant differences by group in the primary and secondary outcome variables. Given the differences observed between groups in pretreatment levels of depression, anxiety and self-esteem, we conducted one-factor ANCOVA_s_ to control the possible effect of these differences in baseline measures. After controlling pretreatment values, no significant effects of group (depression: F (1,50) = 0.620, *p* = 0.435, η^2^_p_ = 0.012; anxiety: F (1,50) = 1.22, *p* = 0.274, η^2^_p_ = 0.024; self-esteem: F (1,50) = 1.07, *p* = 0.307, η^2^_p_ = 0.021 ) or covariables (depression-pre: F (1,50) = 2.69 *p* = 0.107, η^2^_p_ = 0.051; anxiety-pre: F (1,50) = 1.22, *p* = 0.274, η^2^_p_ = 0.024; and self-esteem: F (1,50) = 0.12, *p* = 0.912, η^2^_p_ = 0.000) emerged.

We found a significant group x time interaction effect for depression and anxiety but not for posttraumatic symptoms and self-esteem (Table 2). Post-hoc comparisons revealed significant improvements in depressive symptoms from pretreatment to almost all the measures for both formats, although they showed a different pattern of improvement (Figure 3). The individual format showed a significant decrease in depressive symptoms between pretreatment and posttreatment (*p* = 0.001), which was maintained and then augmented at three-months follow-up, with significant differences between one and three-months follow-up (*p* = 0.019). In the group condition, there was also a significant decrease in depression symptoms from pretreatment to posttreatment (*p* < 0.001) and then from the six-months follow-up, with significant differences between posttreatment and 6- (*p* = 0.002) and 12-months (*p* = −001) follow-ups. Post-hoc comparisons did not reveal significant difference in depression between conditions in any of the times of measure.

Concerning anxiety (Figure 4), in the individual condition, we found a significant decrease in symptom severity from pre-treatment to post-treatment (*p* = 0.010), as well as to all follow-ups (*p* < 0.001 for all comparisons); symptoms showed a progressive decrease over time with significant differences between post-treatment to 6- (*p* = 0.003) and to 12-months follow-ups (*p* < 0.001), and between 1- and 12-months follow-ups (*p* < 0.001). The improvements in the group condition appeared to take much more time to emerge; although there was a progressive decrease in anxiety symptoms, it only reached statistical significance from the 6-months follow-up; specifically, the 6-months measure showed significant differences with posttreatment (*p* < 0.001) and 1-month follow-up (*p* = 0.011), while 12-months follow up showed significant differences with posttreatment (*p* < 0.001), 1- (*p* < 0.001) and 3-months follow-ups (*p* = 0.019). However, post-hoc comparisons did not reveal significant difference in anxiety between conditions in any of the times of measure.

From the clinical point of view, as observed in Table 3, there were important improvements in emotional status, with significant reduction in the percentages of women with possible problems of posttraumatic stress, depression, and anxiety in both intervention formats mainly in the long-term. While no significant differences were found between conditions in the percentage of women with clinically relevant posttraumatic, depressive and anxiety symptoms in the pre-treatment and each of the follow-up measures, individual treatment appears to have a slightly greater effect on symptoms. Specifically, McNemar’s tests revealed that the individual condition promotes significant reduction in the rate of clinically meaningful PTSD symptoms from pretreatment to post-treatment (*p* = 0.035), 1-month (*p* = 0.001), 3-months (*p* ≤ 0.001) and 12-months (*p* < 0.001) follow-ups. In the group condition significant improvements required more time. The proportion of women assigned to the group condition with clinically meaningful scores did not decline significantly from pre-treatment to post-treatment (*p* = 0.424) and from pre-treatment to 1-month follow-up (*p* = 0.118). However, there was a significant reduction of this proportion from pre-treatment to 3- (*p* = 0.002), 6- (*p* = 0.003) and 12-months (*p* = 0.002) follow-ups.

Regarding depression, both conditions promote significant decline in the proportion of participants with clinical meaningful scores from pre-treatment to post-treatment (individual: *p* = 0.012; group: *p* = 0.006), 1-month (individual: *p* = 0.001; group: *p* = 0.001), 3-months (individual: *p ≤* 0.001; group: *p* = 0.002), 6-months (individual: *p ≤* 0.001; group: *p* < 0.001) and 12-months (individual: *p ≤* 0.001; group: *p* < 0.001) follow-ups.

Finally, although individual intervention promotes a significant reduction in clinically meaningful anxious symptomatology from pre-treatment to all other measures (post-treatment: *p* = 0.012; 1-mont: *p* = 0.012; 3-months: *p ≤* 0.001; 6-months: *p* = 0.001; 12-months: *p* < 0.001), group condition only showed a significant decline of this proportion from pre-treatment to post-treatment (*p* = 0.006), 6-months (*p* = 0.022) and 12-months (*p* = 0.022) measures.

### 3.4. Effect of Individual and Group Formats on Social Variables

Finally, to test the effect of both formats of treatment on social issues, the progression in the perception of familiar and social support was analyzed. It is worth mentioning that, in pre-treatment assessment, most women in both conditions showed family support, and to a lesser extent, also social support (Table 1)

Regarding treatment effects, no significant variations were found between pre-treatment and follow-ups in the presence or absence of family support in both the individual and in the group condition. Moreover, as can be seen in Table 3, no significant differences between conditions were found at any time measure.

Conversely, treatment promoted significant improvements on perceived social support. In the individual condition, there were significant differences from pretreatment to posttreatment (*p* = 0.008), 3-months (*p* = 0.039) and 12-months follow-ups (*p* = 0.039). Specifically, among women that did not reported social support at pretreatment, 72.7% had it at posttreatment and 3-months follow-up and 90.9% at 12-months follow-up. In the group condition, the variations in social support only approached significance at a short-term (*p* = 0.007), given the 70% of participants that did not report social support at pretreatment and did it at posttreatment. Otherwise, no significant differences were found when comparing pretreatment reports and all other measures (1-month: *p* = 0.180; 3-months: *p* = 1.00; 6-months: *p* = 1.00: 12-months: *p* = 0.302). Nevertheless, as can be seen in Table 3, no significant differences between conditions were found at any time measure.

## 4. Discussion

This study compares individual vs. group formats of a CBT trauma-focused intervention that has previously shown its effectiveness for women victims of IPV with sub-clinical PTSD symptoms [16]. Further, it specifically analyses the effect of both formats on social issues, which has been claimed to be an advantage for group interventions [13,14]. In this way, it provides valuable information for the design of resources and the implementation of interventions to improve emotional state and psychopathological symptoms in female survivors of IPV.

The data show that the trauma-focused CBT proposed herein (both intervention formats) has a significant effect, with significant reductions in posttraumatic, depression and anxiety symptoms in women that have suffered severe and long-lasting IPV (over 10 years in both groups); for all these variables, the changes emerge at posttreatment and are maintained in the follow-ups up to one year after intervention. In addition, it also achieved significant improvement in self-esteem that becomes significant since 1-month follow-up. Moreover, data show a significant clinical effect with significant decreases in the percentages of women with clinically significant emotional problems [37].

According to Arroyo et al. [10] findings, the effect of both interventions was higher in posttraumatic symptomatology. However, this result contrasts with the recent Cochrane Review by Hameed et al. [38], where, according to the computed effect sizes, it was concluded that, although psychological therapies probably reduce depression and may reduce anxiety symptoms in IPV survivors at medium-term (6 to 12 months), there is no certainty about their beneficial effect on PTSD symptoms. While our findings also point to larger effect sizes in the reduction of depression and anxiety symptoms intensity, the discrepancy with the Cochrane Review [38] regarding PTSD symptomatology may respond to certain methodological differences. First of all, although our treatment combined different techniques, it constituted a trauma-focused intervention and the larger effect on PTSD symptoms is coherent with its main objective. By contrast, the above-mentioned reviews include different types of interventions on the basis of more flexible or broad inclusion and exclusion criteria. While Hameed et al. [38] started their review from a clear clinical definition of the psychotherapy concept, Arroyo et al. [10] considered a wider definition, including some less clinical and structured techniques like yogic breathing or counseling. Moreover, participants in our study and across all studies reviewed by Arroyo et al. [10] were heterosexual females abused by men, whereas Hameed et al. [37] included some studies with a minor proportion of women who report IPV perpetrated by a same-sex partner. In addition, contrary to the face-to-face and brief nature of our intervention and all treatments included by Arroyo et al. [10], Hameed et al. [38] did not establish these restrictions. Nevertheless, our study responds precisely to the need claimed in these reviews of further research with consistent methodologies to increase the evidence-based knowledge about the intervention with IPV survivors.

The treatment accomplished long-term effects, enabling women to handle trauma derived from IPV; moreover, improvements consolidated and even increased over time. These results are in line with those obtained with different versions of this trauma-focused CBT in group format (e.g., [16,20,39,40,41]) and with other CBT in individual format [42] or individual and combined one (i.e., individual + group; Echeburúa et al. [11]), and point that trauma-focused CBT can meet the demands and needs of female survivors of IPV, improving, clinically and significantly, their emotional status.

The good effect of treatment could be limited by the high percentage of dropouts (28.3%), which, however, is similar to that of other interventions (e.g., Kubany et al. [42]: 20% in individual format). As well as the lack of motivation, these high rates may be related to the specific circumstances of these women (i.e., undergoing a transition period with frequent changes of home, work status, etc.). Moreover, the higher percentage of dropouts in the group format (37.71% vs. 20% in individual) may relate to the difficulties for flexibility in the schedule and access to the sessions. Nonetheless, neither this difference, neither differences between formats in the accomplishment of homework nor satisfaction with therapy reached statistical significance, which would support the implementation of the group format.

In this line, and against initial predictions and previous results (cf. Arroyo et al. [10]), there were only marginal significant differences between both delivery formats. While Fernández-Velasco [12], applying this same program to women with PTSD diagnosis, reported that the individual format was superior to group intervention, mainly at 6- and 12-months follow-ups, in this study both formats obtained similar effects, and differences tended to decrease over time, until almost disappearing at 6-months and mostly 12-months follow-ups. Discrepancies between present results and Fernández-Velasco’s [12], who focused on women with PTSD diagnosis, could point that individual format poses some advantages for IPV survivors with a more severe emotional impact (namely, PTSD diagnosis). It should be noted that Echeburúa et al. [11], when comparing individual vs. individual + group formats, found that combined therapy did better than the individual one in PTSD symptoms and impaired functioning at follow-up assessment, partially supporting the beneficial effects of group therapy as adjunctive to individual CBT.

Furthermore, contrary to expectations, the group format did not present any advantage to get family or social support. In fact, neither of the two formats appear to have any effect on family support. Conversely, although no differences were found between formats in social support, the individual treatment promotes significant short- and long-term improvements, whereas, in the group condition, the variations in social support only approached significance in the short-term. Maybe a close therapeutic bond established in the individual treatment might be more powerful than social encouragement provided by the group, which could have a limited effect that disappears once the therapy is finished. In addition, it should be taken into account that women in the present study had a good initial level of social and family support, which could have provoked a “ceiling effect”; the analysis of this element in highly isolated women is thus open to further research.

The study complies with most of the requirements for research on treatments outcomes [19]: a well-trained psychologist delivered the interventions, using manualized protocols, which would facilitate their future application; random assignment of participants to the experimental conditions; in-depth assessment; a fixed number of sessions; and inclusion of long-term follow-up. Nonetheless, some limitations of the study must be pointed out. First, the generalization of results is potentially limited by the fact that all treatments were applied by the same therapist; even though the use of a manualized protocol could contribute to mitigate potential biases. Secondly, the evaluations were not performed by blind interviews. Thirdly, the small sample size, mainly at the follow-ups, could have implications for statistical power. Finally, the study does not include a control group; although a waiting-list control group was initially planned, it was finally discarded since, in the pilot study, none of the women from the waiting-list subsequently entered treatment [34], and due to the ethical considerations of giving women no access to the intervention that they demand and may be a crucial tool for their recovery. Nonetheless, the inclusion of a comparison group with treatment as usual should be considered for the future. Likewise, the inclusion of women who still live with their abuser could be questioned.

All in all, this study has significant clinical implications. It provides additional support of a brief trauma-focused therapy (eight session) that have proved to be effective in the reduction of symptoms and discomfort in IPV survivors with significant posttraumatic symptoms; actually, it has been included as such in several recent reviews on this topic (cf. [10,43,44,45]). Furthermore, since data do not show significant differences between individual and group delivery formats, or in symptoms reduction or in dropouts, group application of this treatment emerges as a therapeutic alternative that could be very helpful due to its cost-effectiveness advantage over individual modalities.

Future research should focus on the adequacy of the format to symptoms severity as well as on the consideration of other variables (e.g., cohabitation with the abuser, availability of social support, motivation for the change, etc.) that would affect the effect of the format; some of them, though included in this study, has not been analyzed due to the small sample size that would affect statistical power and prevent methodologically safe conclusions. Similarly, effect of group format should be tested in IPV survivors that lack social support, since the beneficial social effect of group interaction promoted by this format could arise and gain relevance in isolated women. Moreover, the inclusion of the combined format (i.e., including some individual and some group sessions) could offer a promising path that would deserve attention, considering the individual and group formats not only as alternatives but also as complements.

## 5. Conclusions

Cognitive-behavioral trauma focused intervention for IPV survivor delivered in the group format proved effective in symptom reduction as an individual format, with no significant increase in dropouts. While it did not seem to imply any advantage in social issues, since it would maintain a good cost-effectiveness balance, it would be a relevant alternative for community, social and clinical services, frequently overloaded, allowing for considerable savings in cost, time and efforts. Nevertheless, the careful analysis of each woman needs and circumstances (e.g., schedule availability, motivation for change, difficulties to sharing emotions and problems, isolation or availability of social support, severity of symptoms, etc.) must prevail as a guide for the choice of the delivery format.

## Figures and Tables

**Table 1:** Characteristics of women, violence situation, emotional status, and groups homogeneity at baseline (*n* = 53).

	Individual (*n* = 25)	Group (*n* = 28)	Statistics	*p*	Effect Size
Age in years M (SD)	38.04 (10.53)	40.18 (9.95)	*t* (51) = −0.76	0.451	d = 0.209
Social class *n* (%)			Fisher exact test	0.071	-
Low	6 (24.0)	3 (10.7)			
Medium-low	4 (16.0)	9 (32.1)			
Medium	15 (60)	12 (42.9)			
Medium-high	0 (0.0)	4 (14.3)			
Marital status *n* (%)			Fisher exact test	0.454	-
Single	7 (28.0)	6 (21.4)			
Living with stable partner	3 (12.0)	1 (3.6)			
Married	2 (8.0)	6 (21.4)			
Divorced	13 (52.0)	14 (50.0)			
Widowed	0 (0.0)	1 (3.6)			
Work status *n* (%)			Fisher exact test	0.683	-
Active	5 (20.0)	8 (28.6)			
Unemployed	7 (28.0)	6 (21.4)			
Housewife	3 (12.0)	6 (21.4)			
Disabled	1 (4.0)	0 (0.0)			
Educational *n* (%)			Fisher exact test	0.269	-
Incomplete primary studies	5 (20.0)	1 (3.6)			
Complete primary studies	9 (36.0)	10 (35.7)			
Secondary level	9 (36.0)	12 (42.9)			
University level	2 (8.0)	5 (17.9)			
Lives with aggressor *n* (%)	7 (25.0)	9 (32.1)	*X*^2^ (1,53) = 0.001	0.977	Φ = 0.045
Depends economically on aggressor *n* (%)	8 (32.0)	12 (42.9)	*X*^2^ (1,53) = 0.28	0.596	Φ = 0.112
Duration (years) of maltreatment M (SD)	12.00 (10.89)	10.61 (10.42)	*t* (51) = 0.48	0.636	d = 0.130
Frequency of maltreatment past month *n* (%)			Fisher exact test	0.640	-
Daily	9 (36.0)	15 (53.6)			
Once a week	3 (12.0)	2 (7.1)			
Once a month	5 (20.0)	5 (17.9)			
Has not occurred	8 (32.0)	6 (21.4)			
Type of maltreatment *n* (%)			Fisher exact test	0.080	-
Psychological	11 (44)	6 (21.4)			
Physical	0 (0.0)	1 (3.6)			
Psychological + Physical	12 (48)	19 (67.9)			
Psychological + Sexual	1 (4.0)	0 (0.0)			
Physical + Sexual	1 (4.0)	0 (0.0)			
Physical + Psychological + Sexual	0 (0.0)	2 (7.1)			
Has reported maltreatment *n* (%)	17 (68.0)	15 (53.6)	*X*^2^ (1,53) = 0.62	0.429	Φ = −0.147
Has had to leave home *n* (%)	11 (44.0)	17 (60.7)	*X*^2^ (1,53) = 0.89	0.347	Φ = 0.167
Medical attention *n* (%)	9 (36.9)	10 (35.7)	*X*^2^ (1,53) = 0.00	1.00	Φ = −0.003
Psychiatric/psychological attention *n* (%)	7 (28.0)	13 (46.4)	*X*^2^ (1,53) = 1.20	0.272	Φ = 0.190
Receives medication *n* (%)	13 (52.0)	15 (53.6)	*X*^2^ (1,53) = 0.00	1.00	Φ = 0.016
Legal support *n* (%)	16 (64.0)	12 (42.9)	*X*^2^ (1,53) = 1.60	0.206	Φ = −0.211
Family support *n* (%)	20 (80)	22 (78.6)	*X*^2^ (1,53) = 0.00	1.00	Φ = −0.018
Social support *n* (%)	14 (56)	18 (63.3)	*X*^2^ (1,53) = 0.11	0.738	Φ = 0.085
Suicidal ideation *n* (%)	10 (40.0)	17 (60.7)	*X*^2^ (1,53) = 1.51	0.218	Φ = 0.207
Posttraumatic symptoms (EGS) M (SD) (0–51)	17.56 (6.44)	14.86 (6.51)	*t* (51) = 1.51	0.136	d = 0.041
Depression (BDI) M (SD) (0–63)	32.24 (12.61)	24.18 (14.03)	*t* (51) = 2.08	0.042	d = 0.596
Anxiety (BAI) M (SD) (0–63)	31.56 (11.83)	24.48 (12.54)	*t* (51) = 2.05	0.045	d = 0.563
Self-esteem (Rosenberg’s) M (SD) (10–40)	25.80 (4,06)	28.00 (3.45)	*t* (51) = −3.20	0.002	d = 0.874
Alcohol consumption (CAGE) M (SD) (0–4)	0.08 (0.27)	0.14 (0.44)	*t* (51) = −0.49	0.042	d = 0.136

M = mean; SD = Standard Deviation; d = Cohen’s d; Φ = phi coefficient; EGS = Severity of Posttraumatic Stress Disorder Symptoms Scale; BDI = Beck Depression Inventory; BAI = Beck Anxiety Inventory; Rosenberg’s = Rosenberg’s Self-Esteem Scale; CAGE = Cut-down, Annoyed, Guilty & Eye-opener.

**Table 2:** Means, standard deviations and repeated measures ANOVA statistics for clinical variables (*n* = 53).

Variable	Individual (*n* = 25)		Group (*n* = 28)		ANOVA			
	M	SD	M	SD	Effect	F (1,51)	*p*	η^2^_p_
**PTSD (EGS)**								
Pretreatment	17.56	6.45	14.86	6.51	Group	0.30	0.584	0.006
Posttreatment	8.84	6.99	9.64	6.69	Time	65.15	<0.001	0.561
1 month	4.28	4.00	5.78	6.96	Group × Time	2.08	0.070	0.039
3 months	3.44	3.90	5.78	6.15				
6 months	4.04	3.72	4.86	5.83				
12 months	3.04	3.76	3.96	5.21				
**Depression (BDI)**								
Pretreatment	32.44	12.84	25.03	13.00	Group	0.06	0.801	0.001
Posttreatment	17.36	10.82	17.36	14.48	Time	41.58	<0.001	0.449
1 month	15.28	10.49	15.25	12.13	Group × Time	2.45	0.034	0.046
3 months	10.48	8.68	13.36	10.36				
6 months	9.48	7.30	10.39	10.37				
12 months	8.96	8.84	9.25	9.55				
**Anxiety (BAI)**								
Pretreatment	32.56	13.11	25.36	12.44	Group	0.079	0.780	0.002
Posttreatment	20.72	11.83	22.32	16.19	Time	35.13	<0.001	0.408
1 month	16.40	12.34	18.61	14.06	Group × Time	2.64	0.024	0.049
3 months	13.24	10.88	16.50	14.03				
6 months	10.76	9.21	12.14	8.43				
12 months	8.00	7.23	10.82	9.00				
**Self-esteem (Rosenberg’s)**								
Pretreatment	25.12	4.05	28.28	3.12	Group	3.23	0.078	0.060
Posttreatment	28.68	3.21	28.64	4.18	Time	17.70	<0.001	0.258
1 month	29.40	3.01	31.50	3.40	Group × Time	1.52	0.184	0.029
3 months	30.68	3.14	31.64	5.00				
6 months	31.64	2.39	32.61	3.68				
12 months	30.28	5.47	30.93	6.02				

M = mean; SD = Standard Deviation; η^2^p = partial eta-square; EGS = Severity of Posttraumatic Stress Disorder Symptoms Scale; BDI = Beck Depression Inventory; BAI = Beck Anxiety Inventory; Rosenberg’s = Rosenberg’s Self-Esteem Scale.

**Table 3:** Frequencies and comparisons between conditions in clinically significant symptomology and support (*n* = 53).

	Individual (*n* = 25)	Group (*n* = 28)	Statistics	*p*	Φ
**Posttraumatic symptoms (EGS)***n* (%)					
Pretreatment	15 (60)	12 (42.9)	*X*^2^ (1,53) = 0.94	0.332	−0.171
Posttreatment	6 (24.0)	8 (28.6)	*X*^2^ (1,53) = 0.004	0.948	0.052
1 month	1 (4.0)	5 (17.9)	Fisher exact test	0.196	-
3 months	1 (4.0)	2 (7.1)	Fisher exact test	1.00	-
6 months	0 (0.0)	1 (3.6)	Fisher exact test	1.00	-
12 months	1 (4.0)	2 (7.1)	Fisher exact test	1.00	-
**Depression (BDI)***n* (%)					
Pretreatment	24 (96.0)	23 (82.1)	*X*^2^ (1,53) = 1.33	0.248	−0.218
Posttreatment	15 (60.0)	13 (46.4)	*X*^2^ (1,53) = 0.51	0.476	−0.136
1 month	11 (44.0)	10 (35.7)	*X*^2^ (1,53) = 0.11	0.738	−0.085
3 months	7 (28.0)	11 (29.3)	*X*^2^ (1,53) = 0.33	0.565	0.119
6 months	7 (28.0)	8 (28.6)	*X*^2^ (1,53) = 0.00	1.00	0.006
12 months	5 (20.0)	6 (21.4)	*X*^2^ (1,53) = 0.00	1.00	0.018
**Anxiety (BAI)***n* (%)					
Pretreatment	24 (96.0)	21 (75.0)	*X*^2^ (1,53) = 3.05	0.081	−0.293
Posttreatment	19 (76.0)	17 (60.7)	*X*^2^ (1,53) = 0.80	0.371	−0.163
1 month	15 (60.0)	16 (57.1)	*X*^2^ (1,53) = 0.00	1.00	−0.029
3 months	9 (36.0)	14 (50.0)	*X*^2^ (1,53) = 0.56	0.454	0.141
6 months	10 (40.0)	12 (42.9)	*X*^2^ (1,53) = 0.00	1.00	0.029
12 months	7 (28.0)	12 (42.9)	*X*^2^ (1,53) = 0.70	0.401	0.155
**Family support***n* (%)					
Pretreatment	20 (80.0)	22 (78.6)	X^2^ (1,53) = 0.00	1.00	−0.018
Posttreatment	18 (72.0)	22 (78.6)	*X*^2^ (1,53) = 0.05	0.814	0.076
1 month	18 (72.0)	25 (89.3)	*X*^2^ (1,53) = 1.57	0.210	0.221
3 months	21 (84.0)	20 (71.4)	*X*^2^ (1,53) = 0.58	0.446	−0.150
6 months	18 (72.0)	22 (78.6)	*X*^2^ (1,53) = 0.05	0.814	0.076
12 months	17 (68.0)	18 (64.3)	*X*^2^ (1,53) = 0.00	1.00	−0.039
**Social support***n* (%)					
Pretreatment	14 (56.9)	18 (64.3)	*X*^2^ (1,53) = 0.11	0.738	0.085
Posttreatment	22 (88.0)	24 (85.7)	*X*^2^ (1,53) = 0.00	1.00	−0.034
1 month	19 (76.0)	23 (82.1)	*X*^2^ (1,53) = 0.04	0.833	0.076
3 months	21 (84.0)	18 (64.3)	*X*^2^ (1,53) = 1.72	0.189	−0.223
6 months	20 (80.0)	19 (67.9)	*X*^2^ (1,53) = 0.47	0.491	−0.137
12 months	22 (88.0)	23 (82.1)	X^2^ (1,53) = 0.04	0.833	−0.082

Φ = phi coefficient; EGS = Severity of Posttraumatic Stress Disorder Symptoms Scale; BDI = Beck Depression Inventory; BAI = Beck Anxiety Inventory.

## Data Availability

The data presented in this study are available on request from the corresponding author.
